# Prevalence, risk factors and outcome of congenital anomalies among neonatal admissions in OGBOMOSO, Nigeria

**DOI:** 10.1186/s12887-019-1471-1

**Published:** 2019-04-03

**Authors:** Akinlabi E. Ajao, Ikeola A. Adeoye

**Affiliations:** 1 Department of Epidemiology and Medical Statistics, University of Ibadan, Ibadan, Nigeria; 2 Department of Surgery, Bowen University, Iwo, Nigeria

**Keywords:** Congenital anomalies, Neonates, Prevalence, Risk factors, Outcome, Developing countries

## Abstract

**Background:**

Congenital anomalies (CA) are a major cause of neonatal morbidity and mortality, especially in developing countries. Data on these anomalies are still poorly collated in developing countries. We aimed to assess the prevalence, pattern, risk factors and outcome of congenital anomalies among neonatal admissions in Ogbomoso Town, Nigeria.

**Methods:**

A cross-sectional retrospective study in which a review of the records of all neonates admitted in the neonatal unit of the Bowen University Teaching Hospital, Ogbomoso over a five-year period (January 2012–December 2016) was undertaken. The occurrence rate and pattern of anomalies were determined, while factors associated with the occurrence and outcome of anomalies were calculated with the odds ratio and 95% confidence interval. Data entry and analysis were performed using SPSS version 21.

**Results:**

CA were found in 67 of the 1057 neonatal admissions, giving a prevalence rate of 6.3%. Anomalies of the cardiovascular and digestive systems were the most common. A higher proportion of babies referred from other facilities had CA, and this was found statistically significant. There was no significant association between CA and low birth weight, sex, maternal age or parity. The mortality rate among neonates with CA was 10.4%. Although, CA was associated with reduced risk of neonatal mortality compared to those with other acute conditions, this was not statistically significantly.

**Conclusion:**

CA is a major indication for neonatal admissions in Ogbomoso. There is the need to establish a surveillance system for CA and efforts should be made to raise awareness of the occurrence and risk factors of CA in developing countries.

**Electronic supplementary material:**

The online version of this article (10.1186/s12887-019-1471-1) contains supplementary material, which is available to authorized users.

## Background

Congenital anomalies (CA), or birth defects, are structural, behavioural, functional and metabolic disorders that occur during intrauterine life and can be identified prenatally, at birth or later in infancy [1, 2]. While birth asphyxia, prematurity and infections are the leading causes of adverse neonatal outcomes, congenital anomalies contribute significantly to neonatal morbidity and mortality. An estimated 7.9 million children are born with major congenital anomalies every year [3]. The proportion of global neonatal mortality due to these defects increased from 3% in 2008 to 4.4% in 2013 [4, 5]. Unfortunately, more than 90% of congenital anomalies occur in low and middle income countries (LMICs) [6].

Major CAs are defined as anomalies with significant effect on life expectancy and they occur in 2–3% of live births and in 20–30% of still births [1, 7]. Their prevalence, however, varies with time and geographical location reflecting a complex interaction between genetics and environmental factors [8]. They account for about 3% of live births and 15–30% of paediatric hospitalizations in the United States [7, 9]. In Sub-Saharan Africa, Ndibazza et al. reported a prevalence of more than 7% in Entebbe, Uganda and hospital-based studies from Nigeria have shown prevalence ranging between 0.4 and 11.1% [10–13].

All organ systems within the body can be affected by CA. The musculoskeletal system is the most often affected system in studies that have focused on externally visible anomalies [3, 8, 10, 14, 15]. In other studies, the cardiovascular and gastrointestinal systems have predominated [9, 16–18]. In previous studies from Nigeria, the gastrointestinal system has been the most reported [13, 19, 20].

Understanding the aetiology of CA is important in prevention and in genetic counselling that may help in eradication. Generally, the aetiology of birth defects remains unclear but is thought to be multifactorial. These factors may be genetic (10–30%), environmental (5–10%) or due to multifactorial inheritance (20–35%), while 30–45% are unknown [7]. Infectious agents appear to be the most important environmental factor in LMICs [6]. Implicated maternal factors include age, lifestyle, illnesses during pregnancy, antenatal care, medication use and non-use of peri-conceptual folic acid [1, 7, 14, 17, 21]. Parental consanguinity, previous miscarriages and stillbirths, and inheritable congenital disease are other important factors in the aetiology of CAs [14, 17, 21].

Mortality is very high among major CA in LMICs rising to 20–85% (as against less than 10% in high-income countries) and generally, mortality is higher among infants with CA compared to normal births [6, 22–24]. Ninety-five per cent of deaths among children with CA worldwide occur in LMICs [22]. A significant number of survivors also suffer life-long disabilities, with birth defects accounting for 25.3 to 35.8 million disability-adjusted life years, worldwide [3, 6].

Despite the huge burden of congenital anomalies in LMICs, there is still a dearth of comprehensive data on these conditions as birth defects registries are absent [3]. There is significant under-estimation of CA in LMICs due to non-presentation at health facilities, under-reporting, deficient diagnostic capacity and poor awareness [3, 25]. Prevalence studies are needed to establish baseline rates, demonstrate changes that occur over time and give clues to aetiology [11]. This study assessed perinatal and maternal factors that may be associated with the occurrence of these congenital anomalies. Outcomes of neonatal admissions with congenital anomalies were compared with other neonatal admissions with other acute conditions to assess their relative morbidity and mortality. This study may, therefore, guide policy makers to increase surveillance of these conditions and perhaps raise awareness of the impact of these anomalies within overall neonatal mortality.

We aimed to assess the prevalence, pattern, risk factors and outcome of congenital anomalies among neonatal admissions in a Tertiary Hospital in a semi-urban area, South-West, Nigeria.

## Methods

### Study setting

This study was conducted at the Neonatal Unit of the Bowen University Teaching Hospital (BUTH), Ogbomoso. BUTH is a faith-based tertiary institution, which is located in semi-urban Ogbomoso, in Oyo State, South-West Nigeria. The institution provides multi-specialist care and serves as a major referral center for hospitals within and outside Ogbomoso, including towns in three states. The hospital was initially established as a general hospital in 1907 but was upgraded to a teaching hospital in 2009. The neonatal unit provides care for neonates delivered within and outside the hospital. However, babies requiring ventilator support and other intensive care are referred elsewhere.

### Study design

This was a cross-sectional study, conducted by retrospectively reviewing the medical records of all neonates admitted at the neonatal unit of BUTH over a five-year period between January 2012 and December 2016. Ethical approval for the study was obtained from the institutional research ethics committee. For the purpose of this study, congenital anomalies were defined as structural defects that were present at birth or identified during the neonatal period, either clinically or through investigation modalities. Categorization of these anomalies was then done using the European Surveillance of Congenital Anomalies (EUROCAT) guidelines [26]. Perinatal asphyxia was defined as failure to establish breathing at birth (using the WHO definition) and an APGAR score of less than 7 after 5 min of birth, while those with APGAR score of 3 or less at this time were classified as having severe perinatal asphyxia. Preterm births were defined as deliveries occurring before the completion of 37-week gestation. Obstetric complications were defined as adverse maternal events that are related to pregnancy, such as antepartum haemorrhage and pregnancy induced hypertensive disorders.

Data were collected on neonatal, birth and maternal characteristics, congenital anomaly status and the outcome of the admission were obtained. Information on neonatal characteristics obtained included: sex, age at presentation, gestational age at birth, birth weight, and type of gestation, place of birth and mode of delivery. Maternal factors obtained were age, parity, antenatal care, folic acid use, febrile illness during pregnancy, self-medication and lifestyle variables, such as smoking and alcohol intake during pregnancy. The outcome variables were the status at discharge (discharged, referred, discharged against medical advice and died); and the mortality experience.

### Statistical analysis

Data collected were analyzed using the SPSS version 21. Categorical variables were summarized using frequencies, ratios and proportions, along with the 95% confidence interval. Continuous variables were summarized using mean, standard deviation, or median and interquartile range (IQR). The prevalence rate of CA was calculated as the proportion of neonates with CA among the total number of neonates admitted during the study period. Associations of neonatal characteristics and maternal socio-demographic factors with congenital anomalies were tested using the chi square statistic. Risk factors were assessed using the binary logistic regression, and the odds ratio and 95% confidence intervals were reported. Bivariate Cox regression analysis was used to assess variables that were significantly associated with neonatal mortality at *p* < 0.2, and significant variables were included in a multivariate Cox regression analysis to determine the predictors of 28-day mortality among neonatal admissions at *p* < 0.05. The level of significance was set at *p* < 0.05.

## Results

**Fig. 1:**
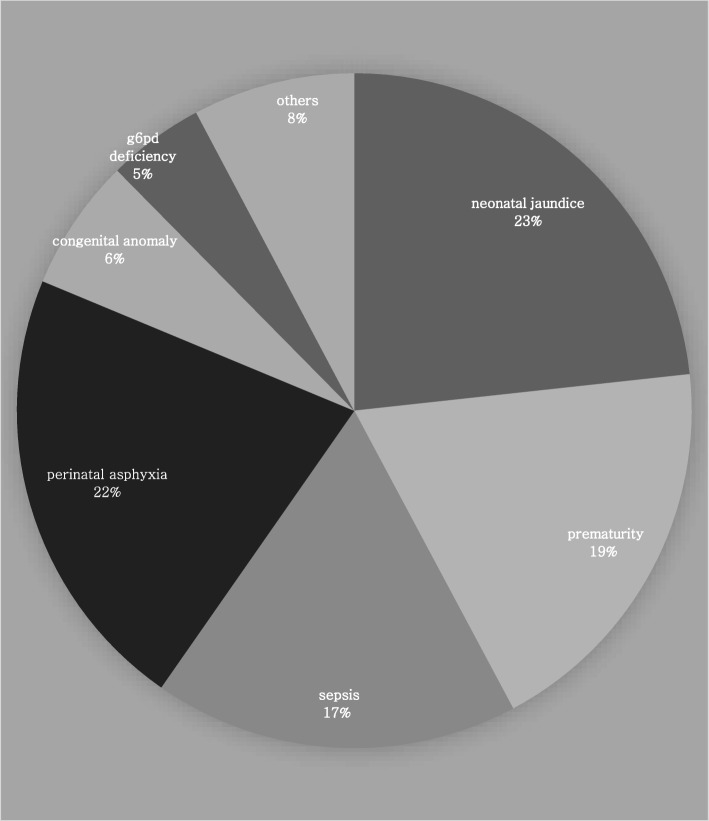
Pie chart illustrating the distribution of neonatal admissions in BUTH between 2012 and 2016

Figure 1 shows the distribution of all neonates admitted based on the disease condition. Sixty-seven (67) of the 1057 admitted neonates had CA during this five-year period giving an overall prevalence rate of 6.3%. The median age at presentation was 2 days (IQR, 6 days) and 36 (53.7%) were males. The mean birth weight was 2.5 (± 0.7) kg, and 58.3% of these neonates had birth weights of 2.5 kg and above. Seventy-five percent of these neonates were delivered via the vaginal route and 38% were delivered preterm (Table 1). Twenty-three patients (34.3%) had associated perinatal asphyxia. The mean maternal age of neonates with congenital anomalies was 29.8 (±5.4) years. A higher proportion of patients referred from peripheral facilities had CA (8.9%) compared to 3.9% of those delivered within the study location, and this difference was statistically significant (*p* < 0.05). The mothers were mainly of the Yoruba ethnic group (89.6%) and 71.6% were domiciled within the town of study.

**Table 1:** Neonatal & maternal socio-demographic factors of study population by congenital anomaly status

Characteristic	Congenital anomaly			
	Yes *n* (%)	No *n* (%)	Total *n* (%)a	*p* value
Sex				
Male	36 (5.9)	576 (94.1)	612 (57.9)	0.475
Female	31 (7.0)	414 (93.0)	445 (42.1)	
Total	67 **(**6.3**)**	990 (93.7)	1057 (100.0)	
Age at presentation				
0-7 days	59 (6.4)	870 (93.6)	929 (87.9)	0.930
8-28 days	8 (6.6)	114 (93.4)	122 (11.5)	
Missing^b^	0 (0.0)	6 (100.0)	6 (0.6)	
Gestational age at birth				
Preterm	13 (5.1)	241 (94.9)	254 (24.0)	0.477
Term	21 (4.0)	503 (96.0)	524 (49.6)	
Missing^b^	33 (11.8)	246 (88.2)	279 (26.4)	
Mode of delivery				
Vaginal	50 (7.1)	659 (92.9)	709 (67.1)	0.228
Abdominal	17 (5.1)	317 (94.9)	334 (31.6)	
Missing^b^	0 (0.0)	14 (100.0)	14 (1.3)	
Place of birth				
Within BUTH	20 (3.9)	494 (96.1)	514 (48.6)	0.001*
Referred	46 (8.9)	473 (91.1)	519 (49.1)	
Missing^b^	1 (4.2)	23 (95.8)	24 (2.3)	
Obstetric complication				
Yes	13 (5.4)	227 (94.6)	240 (22.7)	0.518
No	52 (6.6)	739 (93.4)	791 (74.8)	
Missing^b^	2 (7.7)	24 (92.3)	26 (2.5)	
Birth weight (kg)				
< 1.5	5 (4.8)	100 (95.2)	105 (10.0)	0.420
1.5-2.49	20 (8.5)	216 (91.5)	236 (22.3)	
≥ 2.5	35 (6.6)	495 (93.4)	530 (50.1)	
Missing^b^	7 (3.8)	179 (96.2)	186 (17.6)	
Perinatal asphyxia				
Yes	23 (7.1)	302 (92.9)	325 (30.7)	0.514
No	42 (6.0)	657 (94.0)	699 (66.2)	
Missing^b^	2 (6.1)	31 (93.9)	33 (3.1)	
Maternal age (years)				
≤ 20	4 (12.1)	29 (87.9)	33 (3.1)	0.393
21-35	31 (7.2)	399 (92.8)	430 (40.7)	
>35	4 (4.9)	78 (95.1)	82 (7.8)	
Missing^b^	28 (5.5)	484 (94.5)	512 (48.4)	
Maternal tribe				
Yoruba	60 (6.3)	895 (93.7)	955 (90.4)	0.678
Igbo	3 (9.7)	28 (90.3)	31 (2.9)	
Others	1 (4.2)	23 (95.8)	24 (2.3)	
Missing^b^	3 (6.4)	44 (93.6)	47 (4.4)	
Maternal place of domicile				
Ogbomoso	48 (5.9)	763 (94.1)	811 (76.7)	0.240
Outside Ogbomoso	16 (8.2)	179 (91.8)	195 (18.5)	
Missing^b^	3 (5.9)	48 (94.1)	51 (4.8)	

^a^Column percentage; ^b^Missing data excluded from analysis; *Statistically significant

Anomalies of the cardiovascular system were the commonest anomalies in this cohort, occurring in 14 (20.9%) of the neonates with CAs (Table 2). Three of the neonates with cardiovascular CAs had multiple anomalies and the predominant defect was ventricular septal defect observed in six neonates. This was followed by anomalies of the digestive system occurring in 12 (17.9%), with anorectal malformations being the predominant anomaly affecting four of the neonates. Fifty-three (79.1%) of the anomalies occurred in isolation, while 14 (20.9%) were syndromic.

**Table 2:** Classification of admitted neonates based on congenital anomalies

System	Frequency	%
Nervous system	*n*=4	
Congenital hydrocephalus	2	3.0
Myelomeningocoele	2	3.0
Eye	*n*=2	3.0
Cardiovascular system	*n*=11	
Ventricular septal defect	4	6.0
Atrial septal defect	1	1.5
Patent ductus arteriosus	2	3.0
Heart disease- unspecified^a^	4	6.0
Orofacial cleft	*n*=2	
Cleft lip & palate	2	3.0
Digestive system	*n*=11	
Anorectal malformation^b^	4	6.0
Hirschsprung’s disease	3	4.5
Ileal atresia	1	1.5
Malrotation	1	1.5
Biliary atresia	1	1.5
Oesophageal atresia & trachea-oesophageal fistula	1	1.5
Urinary/Genital	*n*=8	
Hypospadias	1	1.5
Posterior urethral valve	2	3.0
Potter syndrome	1	1.5
Undescended testes	2	3.0
Ambiguous genitalia	2	3.0
Abdominal wall	*n*=5	
Omphalocoele	5	7.5
Limb/Skeletal	*n*=8	
Congenital talipes equinovarus	6	9.0
Genu recurvatum	1	1.5
Polydactyly	1	1.5
Pulmonary	*n*=1	
Laryngomalacia	1	1.5
Other anomalies	*n*=5	
Inguinal hernia	2	3.0
Umbilical hernia^c^	1	1.5
Congenital subcutaneous nodules	1	1.5
Cystic hygroma	1	1.5
Chromosomal abnormalities	*n*=5	
Down syndromeǂ	5	7.5
Multiple anomalies	*n*=5	
Cleft palate, undescended testes & microcephaly	1	1.5
Omphalocoele, VSD & bilateral inguinoscrotal hernia	1	1.5
Microcephaly, ventriculomegaly & VSD	1	1.5
Spina bifida, congenital talipes equinovarus & microcephaly	1	1.5
TEF, microphthalmia & VSD	1	1.5

Categorization based on the European Surveillance of Congenital Anomalies (EUROCAT) guidelines

^a^Clinical diagnosis only, confirmatory studies not done;^b^Two of these babies had an associated ileal atresia or undescended testes; ^c^ Co-existing with glucose-6-phosphate dehydrogenase deficiency; *VSD* ventricular septal defect, *TEF* trachea-oesophageal fistula

Glucose-6-phosphate dehydrogenase (G6PD) deficiency, a metabolic CA, occurred in 50 (4.7%) of the patients. Diagnosis of this condition was established using a qualitative screening test. No quantitative assay had been done on these patients. These neonates were, however, not included in analysis of CAs as the study focused on only structural anomalies.

The practice of self-medication and the use of herbal preparations during pregnancy were associated with higher risks of CAs but this was not statistically significant (*p* > 0.05) (Table 3). Young mothers aged 20 years and less were almost three times as likely as those aged 21–35 years to have babies with CA, while there was a 52% increased risk in those older than 35 years, but this was also not statistically significant. Sex, gestational age at birth, maternal parity and the type of gestation (singleton or multiple) were not significantly associated with the occurrence of congenital anomaly at the 5% level.

**Table 3:** Bivariate analysis showing risk factors associated with the occurrence of congenital anomalies

Characteristic	Odds ratio	95% confidence interval	*p* value
Sex			
Female (Ref)			
Male	0.84	0.51–1.37	0.476
Birth weight (kg)			
<1.5	0.71	0.27–1.85	0.480
1.5–2.49	1.31	0.74–2.32	0.356
≥2.5 (Ref)			
Gestational age at birth			
Preterm	1.29	0.64–2.62	0.478
Term (Ref)			
Maternal age (years)			
≤20	2.69	0.63–11.47	0.181
21–35 (Ref)			
>35	1.52	0.52–4.41	0.446
Maternal parity			
1–2 (Ref)			
3–4	1.13	0.61–2.12	0.692
≥5	2.30	0.92–5.80	0.077
Type of gestation			
Singleton (Ref)			
Multiple	0.65	0.28–1.54	0.326
Antenatal care			
Yes (Ref)			
No	0.78	0.30–2.00	0.606
Self-medication			
Yes	3.02	0.64–14.33	0.163
No (Ref)			
Use of herbal preparation			
Yes	4.08	0.44–37.80	0.215
No (Ref)			
Maternal febrile illness			
Yes	1.26	0.52–3.07	0.615
No (Ref)			
Folic acid use during first trimester			
Yes (Ref)			
No	0.54	0.16–1.83	0.324

**Fig. 2:**
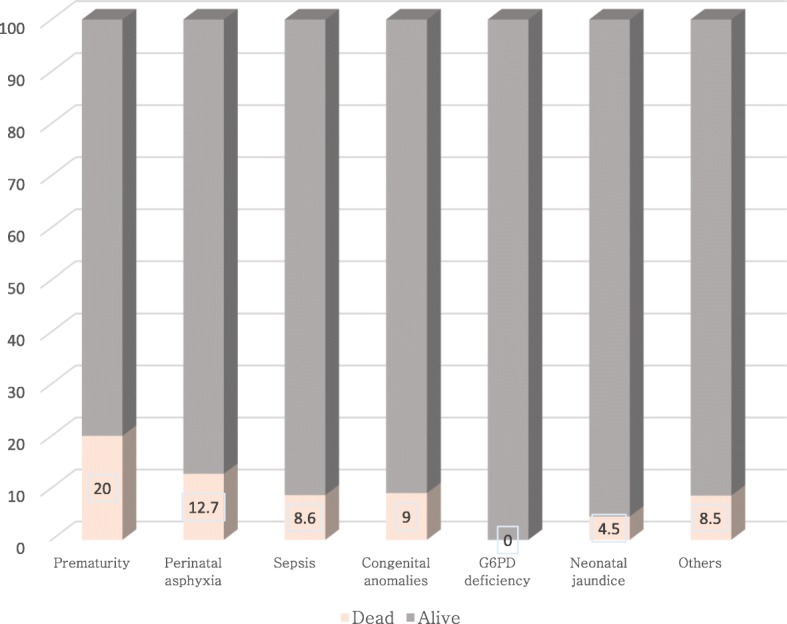
Chart showing proportion of mortality per disease condition

Forty-seven (70.1%) of the neonates were discharged home after initial treatment; six (9%) departed against medical advice; seven (10.4%) were referred and seven (10.4%) died. Neonates with congenital anomalies were more likely to be referred when compared with only 0.1% referred among other neonates (*p* < 0.0001). A total of 111 neonates died during the study period, including seven neonates with CA, giving an overall mortality rate of 10.5%. One hundred and nine of the deaths occurred during the neonatal period, giving a neonatal mortality rate of 10.3%. CA accounted for 5.5% (6/109) of all the neonatal deaths. Prematurity and perinatal asphyxia were the leading cause of neonatal death in this series [Fig. 2].

The neonatal mortality rate (NMR) among patients with CA was 9% (6/67) as shown in Fig. 2. The NMR was, however, higher among those with prematurity and perinatal asphyxia (Fig. 2). The male sex and the presence of a secondary co-morbidity, such as sepsis were associated with an increased risk of neonatal death but the associations were not statistically significant (Table 4). There was no association between CAs and neonatal death after controlling for gestational age at birth, age at presentation, perinatal asphyxia and the presence of co-morbidity in this present study (Table 4). Older age at presentation and multiple gestation were associated with reduced risk of neonatal death and these were found significant at the 10% level of significance. Following multivariate Cox regression analysis, only the presence of severe perinatal asphyxia and gestational age at birth independently predicted the risk of neonatal mortality.

**Table 4:** Bivariate and multivariate analysis of 28-day mortality among neonatal admissions

Characteristic	Crude HR	95% CI	*p* value	Adjusted HR	95% CI	*p* value
Congenital anomaly						
Yes	0.89	0.39–2.02	0.770	0.54	0.13–2.23	0.393
No (Ref)						
Sex						
Male	1.22	0.83–1.80	0.308			
Female (Ref)						
Gestational age at birth (in weeks) ^‡^	0.87	0.82–0.92	< 0.0001^a^	0.86	0.81–0.91	< 0.0001*
Age at presentation (in days) ^‡^	0.93	0.88–0.99	0.015 ^a^	1.05	0.97–1.13	0.199
Birth weight						
<1.5 kg (Ref)						
1.5–2.49 kg	1.04	0.48–2.28	0.913			
≥2.5	1.26	0.62–2.54	0.527			
Type of gestation						
Singleton (Ref)						
Multiple	0.54	0.26–1.10	0.090 ^a^	0.73	0.31–1.72	0.476
Severe perinatal asphyxia						
Yes	3.97	2.66–5.93	< 0.0001^a^	5.41	3.05–9.62	**<** 0.0001*
No (Ref)						
Co-morbidity						
Yes	1.40	0.89–2.20	0.150 ^a^	1.18	0.63–2.19	0.603
No (Ref)						
Maternal parity						
1–2 (Ref)						
3–4	0.87	0.53–1.42	0.575			
≥5	0.58	0.18–1.85	0.357			
Antenatal care						
Yes (Ref)						
No	0.57	0.23–1.41	0.222			

*Statistically significant; ^‡^treated as a continuous variable

## Discussion

Nigeria ranks only second to India in the absolute number of annual neonatal deaths worldwide [4, 27, 28]. CA contribute significantly to this burden, particularly in developing countries [22]. This study was a five-year review of the occurrence, pattern and outcome of CA and its contribution to neonatal mortality in a tertiary health facility in a developing country. The overall prevalence of CA in this study was 6.3%. Our institution receives major referrals from several surrounding communities and this may have accounted for this high yield of anomalies. This demonstrates the magnitude of the burden in developing countries. A high prevalence of CA of 11.1% among hospital admissions was reported by Adeyemo et al in Ibadan, South-West Nigeria [13]. Our finding was similar to the 6.9% reported by Bakare et al in Ile-Ife, which is within the same geographical region as our centre [12]. However, similar studies conducted in the South-East and South-South regions of Nigeria have yielded lower prevalence rates of 2.8 and 0.4%, respectively [10, 11]. The lower rate in the South-South region may have been due to their lower utilization of health facilities for delivery (50.1%), as shown in the NDHS data [29].

Prevalence studies such as this, help to determine baseline rates and to identify changes over time, which are essential in public health planning of preventive and eradication strategies. This study demonstrates the prevalent nature of birth anomalies. However, we may have significantly under-estimated the actual incidence of these anomalies in the general population. Being a tertiary health facility, complicated cases are more frequently seen, while the uncomplicated cases may have been treated in other peripheral facilities. Anomalies that did not require hospitalization were not captured in this study except where they co-existed with conditions requiring immediate treatment. We also did not include abortions and stillbirths in this study. Previous studies have, however, shown that the incidence of CA is higher among stillbirths and abortuses [7, 17, 30].

The most common anomalies were those of the cardiovascular system, followed by those of the digestive system. This finding was similar to those reported in studies from the United States, Lebanon and the United Kingdom, in which larger cohorts were analyzed [9, 18, 31]. Abudu et al. also reported that the cardiovascular system anomaly was the most common anomaly in autopsies of perinatal deaths in Lagos, Nigeria [30]. The gastrointestinal system was the most common system affected in other studies from Nigeria and North-East India [13, 16, 19, 20]. Some other authors have reported predominance of anomalies of the skeletal and nervous system having focused mainly on externally visible anomalies or self-reports from population-based studies [3, 8, 10, 11, 14, 15, 22].

Related to this, however, is the very significant finding of neonates with the functional anomaly of G6PD deficiency. Almost 5 % of all the neonates had this anomaly, which almost equates the 6.3% contributed by all structural anomalies in this study. This is in keeping with earlier studies on G6PD in Nigeria, where incidences of 24 and 5% were reported in males and females, respectively [32]. The high endemicity of malaria in this environment probably plays a significant role as have been previously suggested [33].

The significant association between low birth weight (LBW) and congenital malformations has been well-documented [8, 14, 15, 17, 18]. Although in the present study, we found that LBW (birth weight between 1.5–2.49 kg) was associated with a 30% higher risk, we did not find this statistically significant. The male sex was associated with a 16% reduced risk of being born with a CA despite the slight male preponderance among this cohort. There was, however, no significant association between sex and the occurrence of CAs, in contrast to several studies, which have reported male association [3, 8, 12, 13, 16, 17, 20].

It is generally known that maternal lifestyle may predispose to development of CA in their offspring. The consumption of alcohol, cigarette and certain medications are known teratogenic factors [1, 18, 21]. In the present study, smoking and alcohol intake among mothers was negligible and hence, were not significantly associated with these anomalies. The prevalence of smoking is known to be low among women in developing countries [34]. However, the possible effect of paternal smoking and its secondary effect on birth defects were not explored in the present study. We, however, observed that the consumption of herbal preparations and self-medication during pregnancy were associated with three to four times the risk of anomalies as those who do not, but these were not found statistically significant. This may, however, be a point of Public Health interest as the consumption of these uncertified preparations appear prevalent among pregnant women in developing countries.

We did not find any significant association between CA and maternal age, parity, febrile illness, preterm delivery and type of gestation. The mean maternal age was 29.8 (±5.4) years, which falls within the active reproductive years. Many studies have shown an increase in the incidence of birth malformations with increasing maternal age, particularly in mothers older than 35 years [14–16, 21]. The odds of delivering babies with a CA was, however, higher among women younger than 21 years and older than 35 years in the present study. The association of CAs with older maternal age may not have been found significant in our study because of the relatively smaller number of women above 35 years and the low incidence of reported chromosomal anomalies, which are the anomalies often associated with older maternal age [14]. Although, there was an increasing risk of anomalies with increasing parity, this relationship was not found to be statistically significant from our study (*p* = 0.077). Some studies have shown significant association between multiparity and the occurrence of congenital anomalies but the authors did not control for the effect of maternal age as a confounding factor [8, 17].

Birth defects have been shown to have significant effect on morbidity and mortality [6]. We assessed the outcome of congenital anomalies and their effect on neonatal mortality, while comparing same with those of neonates admitted for other conditions. We further assessed factors that determined survival among neonates and included the CA status in the multivariate analysis to determine its independent effect on survival.

A majority of neonates (70%) with birth defects were discharged home after initial management in our facility. The overall mortality rate among neonates with CA was 10.4%. This was almost comparable to the 16.9% observed in Ibadan [13]. Mortality from CA accounted for 5.5% of all neonatal mortalities that occurred during this five-year period. This was higher than that reported by Lawoyin et al in South-West Nigeria, where they investigated the perinatal factors associated with neonatal mortality and found a 3.1% contribution by congenital abnormalities [35]. The latter study was, however, a community-based study and the authors relied on self-reports and reports from health attendants where medical records were not available. This may have resulted in a lower yield as they also reported that 34.4% of the neonatal deaths were of unknown cause. Our finding was also similar to the study by Liu et al that reported that congenital anomalies were the fourth leading cause of global neonatal mortality after preterm birth, intra-partum complications and sepsis [5].

The male sex was associated with a higher risk of mortality compared to female neonates. As expected, the presence of co-morbidity increased the risk of neonatal death but did not independently predict the risk. Each additional week of gestation at birth, however, was associated with a 14% reduced risk of neonatal death. Severe perinatal asphyxia increased the risk of neonatal mortality by over five times (Table 4). After eliminating the effect of these other confounders, the CA status did not predict the risk of death.

### Limitations

This was a retrospective review and therefore, had the inherent biases associated with this kind of study. We were unable to retrieve all case files of neonatal admissions; and many of those retrieved, had inadequate information on the perinatal characteristics of the neonates and the maternal risk factors. We could not capture live births that died or were referred without official registration and opening of a case file in our hospital. Furthermore, this was a hospital-based study and may not be generalizable to the general population.

## Conclusion

Congenital anomalies account for 6% of neonatal hospital admissions in Bowen University Teaching Hospital, Ogbomoso, South-West Nigeria. There is the need to establish a surveillance system for CA in Nigeria to determine their true prevalence, pattern and aetiology in the general population. Because of the high frequency of metabolic disorders in this study, we recommend newborn screening for G6PD deficiency. Efforts should also be made to raise awareness of the occurrence and risk factors of birth defects through health education and advocacy. Improvement in obstetric care, prenatal diagnosis of structural anomalies and early neonatal intensive care is also advocated as these can improve survival in this group of neonates.

## Additional files

Additional file 1: Spreadsheet of study data in excel format. (XLS 1146 kb)

Additional file 2: Spreadsheet of study data in .dat format. (DAT 147 kb)

## Abbreviations

BUTH: Bowen University Teaching Hospital
CA: Congenital anomaly
G6PD: Glucose-6-phosphate dehydrogenase
LMIC: Low- and middle-income country
NDHS: Nigerian demographic and health survey
NMR: Neonatal mortality rate
SPSS: Statistical package for social sciences
